# Mutations in *mexT* bypass the stringent response dependency of virulence in *Pseudomonas aeruginosa*

**DOI:** 10.1016/j.celrep.2024.115079

**Published:** 2024-12-20

**Authors:** Wendy Figueroa, Adrian Cazares, Eleri A. Ashworth, Aaron Weimann, Aras Kadioglu, R. Andres Floto, Martin Welch

**Affiliations:** 1 Department of Biochemistry, University of Cambridge, Cambridge CB2 1QW, UK; 2 Victor Phillip Dahdaleh Heart & Lung Research Institute, Department of Medicine, University of Cambridge, Cambridge, UK; 3 Wellcome Sanger Institute, Wellcome Genome Campus, Hinxton, Cambridge, UK; 4 Department of Clinical Infection, Microbiology & Immunology, University of Liverpool, Liverpool L69 7BE, UK; 5 Molecular Immunity Unit, University of Cambridge, Department of Medicine, MRC-Laboratory of Molecular Biology, Cambridge, UK; 6 Cambridge Centre for Lung Infection, Papworth Hospital, Cambridge, UK

## Abstract

*Pseudomonas aeruginosa* produces a wealth of virulence factors whose production is controlled via an intricate regulatory systems network. Here, we uncover a major player in the evolution and regulation of virulence that enhances host colonization and antibiotic resistance. By characterizing a collection of mutants lacking the stringent response (SR), a system key for virulence, we show that the loss of the central regulator MexT bypasses absence of the SR, restoring full activation of virulence pathways. Notably, *mexT* mutations were associated with resistance to aminoglycosides and the last-resort antibiotic, colistin. Analysis of thousands of *P. aeruginosa* genomes revealed that *mexT* mutations are widespread in isolates linked to aggressive antibiotic treatment. Furthermore, *in vivo* experiments in a murine pulmonary model revealed that *mexT* mutants display a hypervirulent phenotype associated with bacteremia. Altogether, these findings uncover a key regulator that acts as a genetic switch in the regulation of virulence and antimicrobial resistance.

## Graphical abstract

**Graphical abstract:**
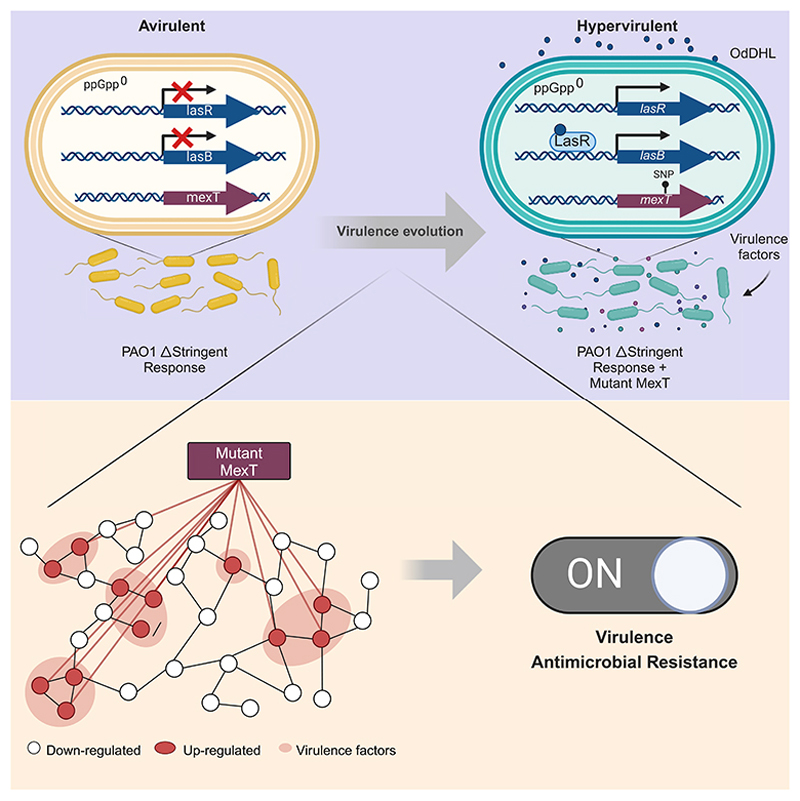

## Introduction

The ability of pathogens to initiate and maintain infections depends on the production of virulence factors. These molecules are associated with, e.g., tissue degradation, iron acquisition, biofilm formation, and evasion of the immune response.^1^ They are energetically costly for the cell to make, and, as a consequence, pathogens have evolved robust regulatory systems to control their production. A well-characterized example is quorum sensing (QS), a mechanism of cell-to-cell communication that is often intimately linked with the control of virulence factor production. Here, as the population cell density increases, individual cells collaborate to ensure the concerted production of virulence factors, thereby facilitating infection by overwhelming the host immune response.^2^

A particularly clear example of how QS controls virulence factor production is seen in *Pseudomonas aeruginosa*. This is an opportunist that has been designated by the World Health Organization as a “priority pathogen” against which new treatments are urgently needed. Unlike many other pathogens, *P. aeruginosa* is a “professional secretor,” and causes tissue damage by secreting a welter of exceptionally active proteases, lipases, and toxins.^3^ The nutrients released as a consequence of tissue degradation are used by the cell for growth; virulence is merely a means to an end of acquiring these. The production and secretion of *P. aeruginosa* virulence factors are controlled by three inter-connected QS pathways, known as the *las, rhl*, and *pqs* sub-systems.^4^ However, a number of other regulatory systems are also known to impinge on virulence factor production.^5,6^ In particular, it was recently reported that the stringent response (SR), a system used by bacteria to sense nutrient limitation, can re-direct cellular resources toward the expression of virulence-associated pathways, including QS.^5,6^ In the SR, nutrient limitation is sensed by two gene products; the ribosome-associated RelA protein and the cytoplasmic SpoT protein, both of which subsequently synthesize a stress alarmone, (p)ppGpp. In most organisms studied to date, the alarmone binds to RNA polymerase, reprogramming it to increase the expression of carbon and nitrogen scavenging pathways and to decrease the expression of genes involved in macromolecular biosynthesis.^7^

Recent studies have shown that the regulatory networks that control virulence factor production have a hierarchical structure and that the SR is positioned at the top of this.^6^ In *P. aeruginosa*, the SR modulates QS by activating the *rhl* and *las* sub-systems and repressing the *pqs* sub-system.^6^ Furthermore, mutants defective in the SR display decreased cytotoxicity toward human epithelial cells and decreased hemolytic activity.^8^ These findings suggest that the SR may be essential for virulence, and that the population needs to be both quorate *and* starving in order to elicit optimal virulence factor production. This “coincidence circuit” makes good economic sense. After all, why invest in producing and secreting energetically costly resources such as virulence factors if the cell is still awash with nutrients?

In this study, we investigated the hierarchical structure that controls virulence in *P. aeruginosa*. Starting with an avirulent mutant deficient in the top tier of regulation (an SR-null mutant), we use a combination of genetic epistasis, genomics, transcriptomics, genome engineering, and an *in vivo* pulmonary infection model to show that loss-of-function mutations in a central transcriptional regulator, *mexT*, bypass the SR requirement and drive the emergence of hypervirulence. This reactivation of virulence phenotypes is accompanied by transcriptional “rewiring” and leads to the restoration of QS. Furthermore, we show that mutations in *mexT* elicit resistance against aminoglycosides and colistin. This was unexpected because MexT has not previously been linked with resistance to these classes of antibiotic, both of which are widely used to treat *P. aeruginosa* infections. We also show that mutations in *mexT* are common and are preferentially associated with *P. aeruginosa* cystic fibrosis (CF) isolates. In many cases, these mutations affect residues close to the predicted ligand-binding pocket of the regulator, leading to its loss of function. Finally, we demonstrate that MexT mutants are hypervirulent in a mouse model of pulmonary infection. Our findings highlight the remarkable regulatory plasticity of *P. aeruginosa* and indicate that the mutational status of MexT plays a key role in bacterial physiology, linking both virulence and antimicrobial resistance.

## Results

### The SR dependence of virulence can be bypassed by secondary mutations

**Figure 1:**
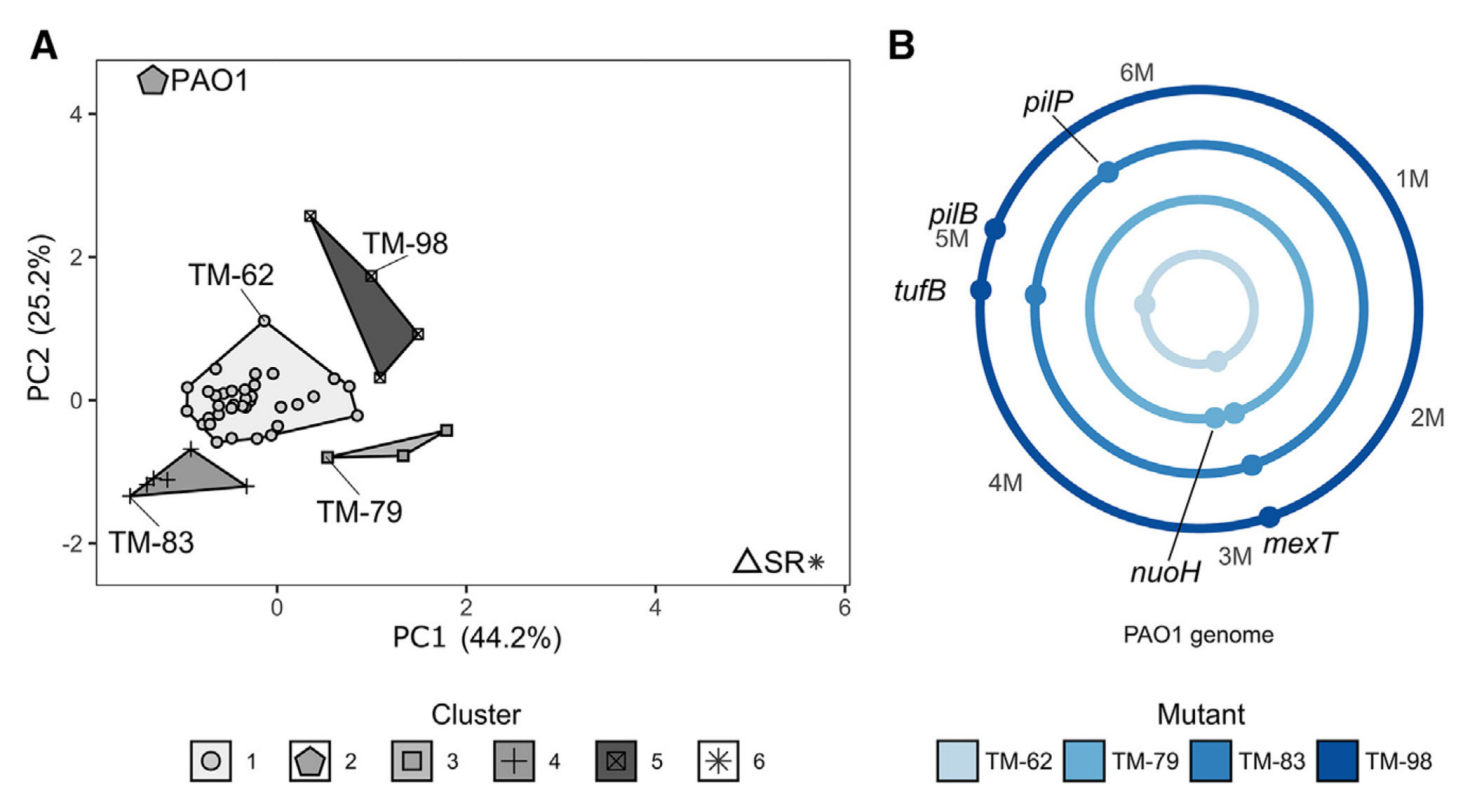
Phenotypic and genotypic characterization of the ΔDSR bypass mutants (A) The virulence phenotypes in a collection of Gm^R^ *P. aeruginosa* bypass mutants were segregated using principal-components analysis (PCA). Data clusters were then defined using k-means analyses. Each k-means cluster is depicted using a different shade of gray, and the mutants belonging to each cluster are delineated using different symbols. The parental mutant lacking the SR (the ΔDSR mutant, *) and the wild-type strain (PAO1, ⬟) are included for reference. The bypass mutants (designated TM-XX) from each cluster selected for genome sequencing are indicated. (B) Mutations identified in representative bypass mutants. Each shade of blue represents the genome of a different mutant. Dots on the circles indicate the genomic position where mutations were found. Numbering is in megabase pairs (M).

A *P. aeruginosa* SR-deficient mutant (ΔSR, containing deletions in *relA* and *spoT*) was unable to produce secreted protease (Figure S2). To look for genes that could restore virulence factor production in this genetic background, we carried out a transposon mutagenesis (using pTnMod-OGm^9^), screening for gentamicin-resistant (Gm^R^) mutants that displayed restored secreted protease production on skim-milk agar plates. From ca. 120,000 mutants screened, we identified 50 Gm^R^ mutants in which secreted protease production had been re-acquired. This collection of protease-producing “bypass mutants” was further characterized by looking for restoration of other virulence-associated phenotypes such as siderophore production, biofilm formation, and swarming motility. The resulting virulence profiles were then analyzed using principal-components analysis (PCA) and segregated into clusters, defined using k-means analyses. Inclusive of the wild-type progenitor strain (PAO1) and of the ΔSR mutant, six distinct clusters were resolved (Figure 1A). Most of the bypass mutants were clearly more similar to PAO1, which is SR proficient, than to the avirulent ΔSR mutant (Figures 1A and S1; Data S1). These data suggest that virulence can be restored independent of the SR.

To determine the genetic basis for the observed restoration of virulence factor production in the Gm^R^ ΔSR mutants, we sequenced the genome of a representative mutant from each cluster (isolates TM-62, TM-79, TM-83, TM-98, and also the genome of the progenitor strains, ΔSR and PAO1) (Figure 1A). Interestingly, and in spite of their robust Gm^R^ phenotype, none of the protease^+^ mutants contained a Tn insertion. However, and relative to the ΔSR progenitor, all of the bypass mutants contained SNPs and/or indels. TM-62, TM-83, and TM-98 all carried a mutation leading to a V21→L substitution in the translation elongation factor encoded by *tufB*. Additional SNPs and indels were found in TM-83 (a 15-bp in-frame deletion in *pilP*) and in TM-98 (a missense mutation, G331→V, in *pilB*) (Figure 1B; Table S1). TM-79 had a 12-bp deletion in the NADH dehydrogenase encoded by *nuoH*. Most notably, though, all the sequenced mutants carried missense mutations in *mexT*, which encodes a transcription factor (Figure 1B). In TM-62, TM-83, and TM-98 the mutation led to a V130→F substitution in the protein, whereas in TM-79 the mutation led to a G207→S substitution.

### Loss of MexT is responsible for SR-independent virulence-associated phenotypes

Given that mutations in *mexT* were a common link among the isolates displaying restored virulence factor production, we prioritized this gene for further investigation. A recent study by Kim et al. reported the structure of the C-terminal regulatory domain of MexT.^10^ This revealed the presence of a putative ligand-binding pocket. Interestingly, despite their separation in the protein sequence, both of the substitutions found in the bypass variants (V130F and G207S) mapped to residues close to this putative binding pocket. This suggested that substitutions in this region of the protein may have a substantial functional impact, possibly even abolishing the protein’s function. To test this further, and to confirm that it was the mutations in *mexT* that drove restoration of virulence in the ΔSR background, we engineered the *mexT*^V130F^ mutation into a “clean” ΔSR progenitor background and also into the wild-type strain (yielding strains ΔSR-mMexT [ΔSR *mexT*^V130F^] and PAO1-mMexT [PAO1 *mexT*^V130F^], respectively). Additionally, to test whether these mutations might lead to loss of function in the encoded *mexT*, we also deleted the gene.

**Figure 2:**
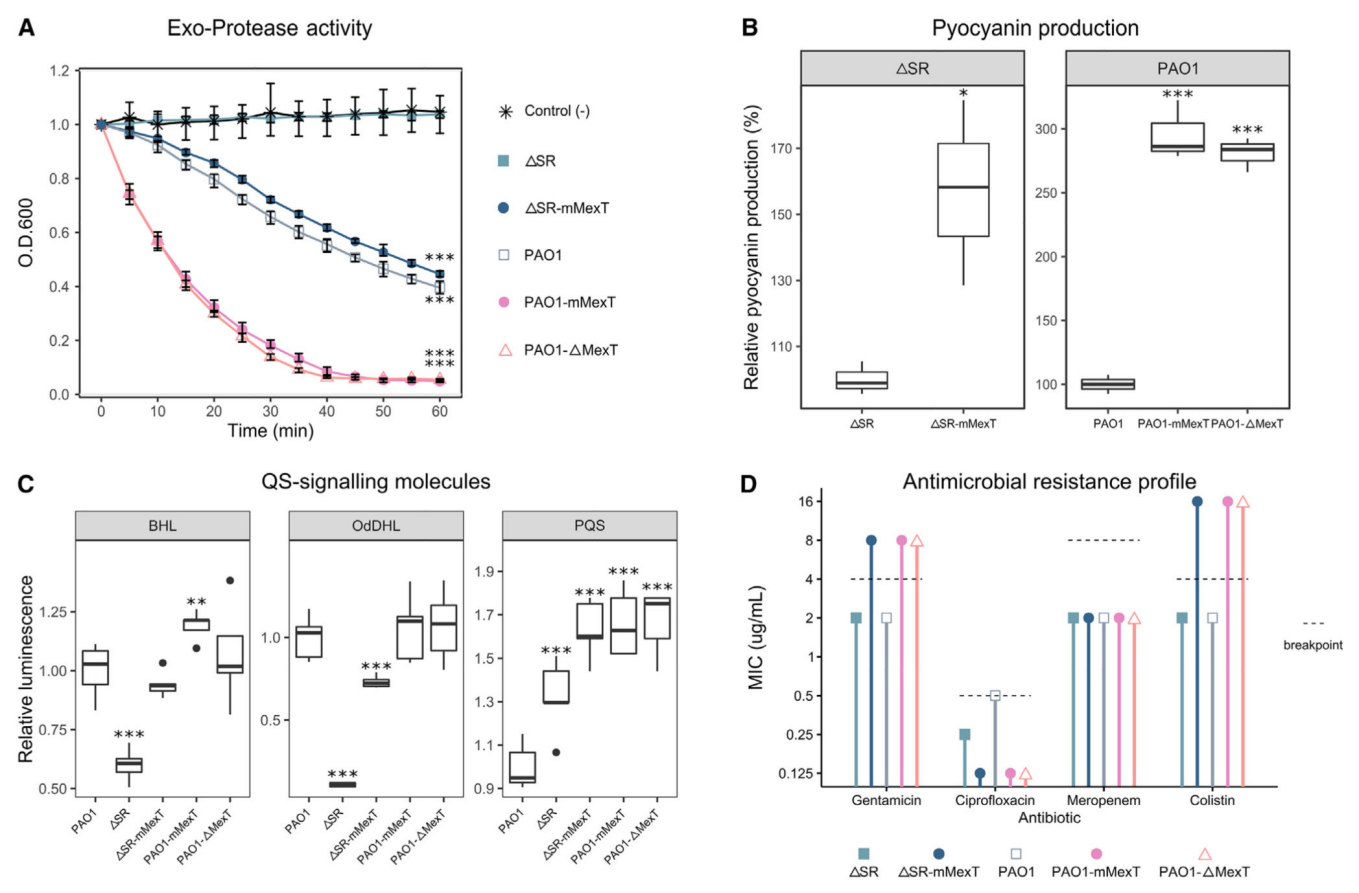
The *mexT*^*V130F*^ mutation leads to increased production of secreted protease, pyocyanin, QS molecules, and elevated antimicrobial resistance (A) The figure shows the proteolytic activity of supernatants from overnight cultures of the parental strains and the indicated *mexT* mutants (mMexT is the *mexT*^V130F^ mutation and Δ*mexT* is the deletion mutant of *mexT*). The degradation of casein in skimmed milk due to exo-protease activity in the supernatants leads to a decrease in the optical density 600 nm (OD_600nm_) compared with the negative control (Lysogeny Broth broth alone). The greater the decrease, the greater the exo-protease activity. Data are represented as mean ± SEM. (B) Pyocyanin production by the *mexT* mutants compared with their progenitor strains. (C) Box-and-whisker plots showing the production of the indicated QS-signaling molecules in cultures of the indicated *mexT* mutants relative to production of the same molecules in cultures of PAO1. (D) Minimum inhibitory concentration (MIC) of antibiotics against the indicated *mexT* mutants and progenitor strains. Dotted lines indicate breakpoints according to the European Committee on Antimicrobial Susceptibility Testing (EUCAST). All experiments were performed in triplicate (*n* = 3). Where they are used, the asterisks denote statistically significant differences based on Welch’s t test, using a Bonferroni correction (*p < 0.05, **p < 0.01, ***p < 0.001).

Subsequent characterization of the engineered mutants revealed that the restoration of virulence-associated phenotypes in the bypass mutants was indeed associated with the mutations in *mexT*. As expected, the wild-type strain (PAO1) displayed robust exo-protease activity, whereas this activity was negligible in the ΔSR mutant (Figure 2A). By contrast, exo-protease activity in the ΔSR-mMexT strain was essentially the same as that in the SR-proficient wild type, PAO1 (Figure 2A). Strikingly, when the endogenous *mexT* was replaced with the *mexT*^V130F^ allele in PAO1 (PAO1-mMexT), exo-protease activity was enhanced even further. A similar enhancement of exo-protease activity was seen when the *mexT* gene was deleted (PAO1Δ*mexT*), suggesting that the V130F substitution likely leads to loss of function in the encoded protein.

The exo-protease data led us to wonder whether the *mexT*^V130F^ and Δ*mexT* mutations would also affect other virulence traits. We therefore investigated their impact on production of the characteristic blue-green pigment made by *P. aeruginosa*, pyocyanin. As shown in Figure 2B, introduction of the V130F mutation into *mexT* led to a large increase in pyocyanin production in both the ΔSR and the PAO1 backgrounds, compared with their respective isogenic progenitors. Consistent with the exo-protease data, the deletion of *mexT* had a similar effect, greatly increasing pyocyanin production.

Virulence factor production is controlled by *N*-acylhomoserine lactone (AHL)- and *Pseudomonas* quinolone signal (PQS)-dependent QS in *P. aeruginosa*.^11^ However, AHL-dependent QS signaling is significantly depressed in the ΔSR mutant. We therefore reasoned that the restoration of virulence phenotypes in the *mexT* mutants might be due to the reactivation of these QS systems. Consistent with earlier reports,^6^ we found that production of the AHL signaling molecules, *N*-butanoyl-L-homoserine lactone (BHL, produced by the *rhl* QS sub-system) and *N*-3-oxo-dodecanoyl-L-homoserine lactone (OdDHL, produced by the *las* QS sub-system) was much lower in the ΔSR strain compared with SR-proficient strains (Figure 2C). However, BHL and OdDHL production was fully restored to wild-type levels in the ΔSR *mexT*^V130F^ strain (Figure 2C). These data indicate that the restoration of virulence-associated phenotypes in this mutant is indeed linked with the restoration of QS. Interestingly, the PQS signaling molecule was increased in the ΔSR mutant (cf. the wild type) and was increased still further in the ΔSR-mMexT, PAO1-mMexT, and PAO1Δ*mexT* strains. These data indicate that PQS production is regulated by a complex interplay involving both the SR and MexT.

### Loss of MexT is associated with aminoglycosides and colistin resistance

Several studies have reported a negative correlation between virulence and antimicrobial resistance.^12^ Nevertheless, it should be borne in mind that the *mexT*-associated bypass mutants identified in the current study were originally isolated because they displayed (1) elevated production of secreted protease and (2) resistance to an aminoglycoside antibiotic, gentamicin. The latter was particularly surprising since *mexT* has not previously been linked with resistance to aminoglycosides. However, MexT is an activator of *mexEF*-*oprN* expression, although the encoded pump exports fluoroquinolone antibiotics, not aminoglycosides. Mutations in *mexT* should, therefore, increase susceptibility to fluoroquinolones such as ciprofloxacin but have no impact on aminoglycosides. In addition to MexT, the SR itself has also been linked with antibiotic tolerance and resistance in diverse bacterial species^13^; consequently, SR-deficient mutants are generally more susceptible to antimicrobials. This prompted us to explore the antimicrobial susceptibility profile associated with the *mexT*^V130F^ allele and the Δ*mexT* mutation in more detail (Figures 2D and S3). Consistent with the role of MexT in stimulating *mexEF*-*oprN* expression, strains containing loss-of-function mutations (*mexT*^V130F^ or Δ*mexT*) displayed increased susceptibility to ciprofloxacin. As expected, susceptibility to carbapenems—which are not a substrate of the MexEF-OprN pump—was unaffected (Figure 2D; Table S2). By contrast, strains containing the *mexT*^V130F^ allele or Δ*mexT* mutation showed uniformly higher resistance (cf. their progenitors, ΔSR and the wild type) to aminoglycoside antibiotics. Hence, the loss of MexT function, either through the V130F substitution studied here or as a consequence of deleting the entire open reading frame (ORF), leads to elevated aminoglycoside resistance. Unexpectedly, the *mexT*^V130F^ and Δ*mexT* strains also displayed an 8-fold increase in minimum inhibitory concentration (MIC) of the “last-resort” antibiotic, colistin (cf. the ΔSR or PAO1 progenitors). All of these antimicrobial resistance (AMR) phenotypes could be fully complemented by provision of the wild-type *mexT* gene *in trans* to the PAO1Δ*mexT* mutant (Table S3). By contrast, complementation of the PAO1Δ*mexT* mutant with plasmid-borne *mexT*^V130F^ did not restore susceptibility to gentamicin and colistin (Table S3).

### The functional state of MexT is more important than the SR for virulence pathways

**Figure 3:**
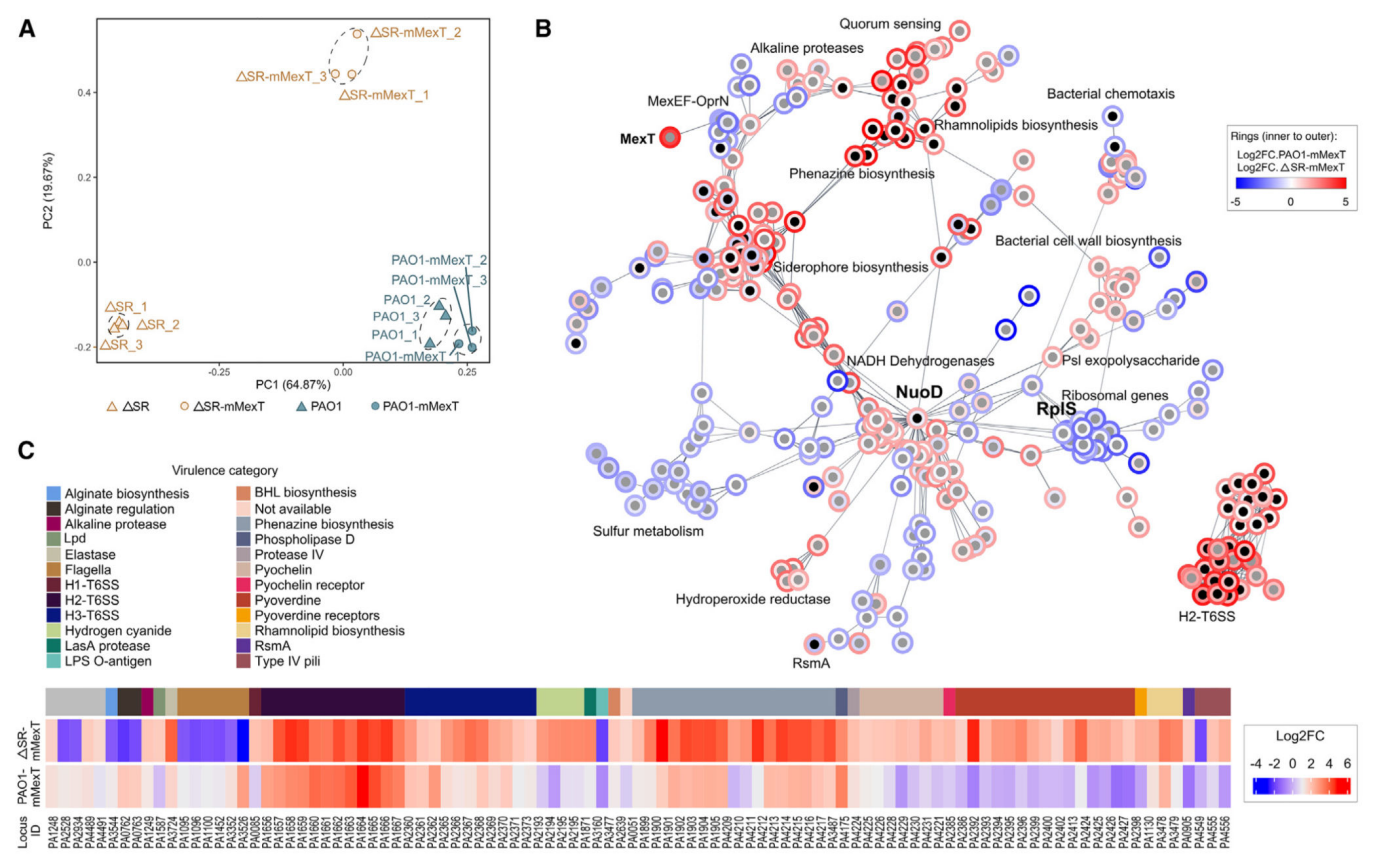
Transcriptional rewiring of *mexT* mutants (A) PCA of the RNA-seq data. Dashed lines delineate the biological replicates of each analyzed strain (*n* = 3). Note that PC1 and PC2 collectively account for ca. 84% of the total variation in the datasets. (B) Network analysis of differentially expressed genes based on Search Tool for the Retrieval of Interacting Genes/Proteins (STRING) functional interactions. The two largest connected component clusters are shown. Each gene transcript (represented as nodes in the network) has two rings; the inner ring shading represents the log_2_ fold change in PAO1-mMexT compared with PAO1, and the external ring shows this value for ΔSR-mMexT compared with ΔSR (note that, for most transcripts, the rings are highly concordant). Blue coloration denotes downregulation, and red indicates upregulation. Black dots indicate genes that encode known virulence factors. (C) Heatmaps of the virulence-associated transcripts that were differentially expressed (log_2_FC ± 1.5, adjusted *p* <0.05) in strains containing the *mexT*^V130F^ allele cf. the corresponding progenitor strain. The colors (from blue to red) indicate the log2 fold change of the specific gene (log2FC from −4 to 6, indicative of a fold change of 0.06–64). Locus tags are shown at the bottom of the panel, and the (color-coded) virulence category for each transcript is shown (e.g., the H2-T6S is shown in dark purple, whereas phenazine biosynthesis genes are shown in gray).

We next investigated how the loss of MexT function driven by the *mexT*^V130F^ mutation was able to bypass the SR-dependency of virulence factor production and whether it had any impact on the transcription of genes associated with aminoglycoside or colistin resistance. To do this, we used RNA sequencing (RNA-seq) to compare the transcriptome of the ΔSR mutant with that of the ΔSR-mMexT strain and of PAO1 with PAO1-mMexT. PCA of the samples revealed that the biological replicates formed discrete clusters, indicating that the most significant transcriptional variation was between the clusters and not within them. Strikingly, the transcriptome profile of the ΔSR-mMexT strain was substantially different from that of its parental strain (ΔSR), moving along PC1 closer to the wild-type background (Figure 3A).

Differential expression analyses of the *mexT* mutants compared with their respective parental strains revealed that 588 genes/ORFs were differentially expressed in the ΔSR-mMexT cf. ΔSR strains (308 upregulated and 280 downregulated), whereas 37 genes/ORFs were differentially expressed in PAO1-mMexT cf. PAO1 (25 upregulated and 12 downregulated) (Figures 3B and S4; Data S2 and S3). Network analyses of the data revealed several functionally connected clusters, with members of each network sub-cluster tending to be differentially expressed in a uniform direction (up- or downregulated) in the ΔSR and PAO1 backgrounds (Figure 3B, full network in Figure S5). As expected, transcripts encoding the MexEF-OprN efflux pump (which is known to be positively regulated by MexT) were downregulated in the *mexT* mutants, consistent with the predicted loss of known MexT function. However, and unexpectedly given its loss-of-function phenotype, we found that *mexT* itself was greatly upregulated in the ΔSR-mMexT and PAO1-mMexT strains. Moreover, we also noted a sub-cluster of downregulated ribosomal transcripts, even in the ΔSR background. This was unexpected because, in most bacteria, ribosome biosynthesis is downregulated during the SR (Figure 3B). Additionally, a plethora of transcripts corresponding to genes encoding proteins of unknown function were downregulated in the presence of the *mexT*^V130F^ mutation in both of the tested genetic backgrounds.

Interestingly, several of the more highly populated clusters in the functional network were linked with virulence and were found to be upregulated in both the ΔSR mutant and the wild type in the presence of the *mexT*^V130F^ mutation. Most obviously, a cluster comprising the H2 type 6 secretion system (H2-T6SS) genes and its activator *sfa2* were upregulated. Additionally, expression of the T6SS-associated phospholipase D was increased in both mutants. Some virulence factors displayed context-specific modulation. For example, transcripts associated with phenazines, pyoverdine, pyochelin, exo-proteases, and rhamnolipids were considerably upregulated in the ΔSR-mMexT strain but not in PAO1-mMexT, suggesting that the regulation of virulence may be different in the absence of a functional SR (Figure 3C). Additionally, genes previously linked with increased bacterial survival in lungs and blood were upregulated in the presence of the *mexT*^V130F^ mutation. For example, *loxA* (lipoxygenase) was expressed 4.5-fold more in PAO1-mMexT compared with PAO1, *cbpD* (a lytic polysaccharide monooxygenase) was upregulated ~14.5-fold in ΔSR-mMexT compared with the ΔSR progenitor, and *chiC* (a chitin-binding protein associated with acute infection) was almost 50-fold more expressed in ΔSR-mMexT compared with ΔSR.

Transcripts associated with O-antigen biosynthesis were downregulated in both strains carrying the *mexT*^V130F^ mutation (cf. their respective progenitors). This may explain the elevated resistance to colistin associated with the *mexT*^*V130F*^ mutation since loss-of-function mutations in O-antigen biosynthetic genes have previously been linked with this phenotype.^14^ As outlined in the section “discussion,” changes in the expression in the O-antigen and lipopolysaccharide (LPS) biosynthetic machinery likely contribute to the elevated resistance to aminoglycosides. Similarly, the increased ciprofloxacin susceptibility associated with the *mexT*^*V130F*^ mutation is likely attributable to the lower expression of *mexEF*-*oprN*.

To gain more insights into the main pathways influenced by the absence of MexT, we carried out a network topology analysis (Data S4). We found that transcripts encoded by genes with the highest closeness centrality, radiality, and eccentricity (indicative of a central role in the network^15^) were associated with the *nuoA-N* operon (especially *nuoD*, which encodes NADH dehydrogenase) and the *rpl* genes (encoding the 50S ribosomal proteins), particularly *rplS* (Figure 3). Additionally, a network-enrichment analysis revealed that all enriched Gene Ontology (GO) molecular functions were related to NADH dehydrogenase activity (Data S5). Interestingly, the *nuo* genes, which were upregulated in the absence of MexT, have previously been associated with virulence.^16^

Taken together, our data indicate that MexT and the SR affect distinct but overlapping virulence pathways and that the functional state of the MexT protein (active, or not functional) is even more important than the SR for control of cellular pathways leading to the production of virulence factors since, even in the absence of the SR, they can be efficiently activated.

### Mutations in *mexT* are common in *P. aeruginosa* isolates from people with CF

**Figure 4:**
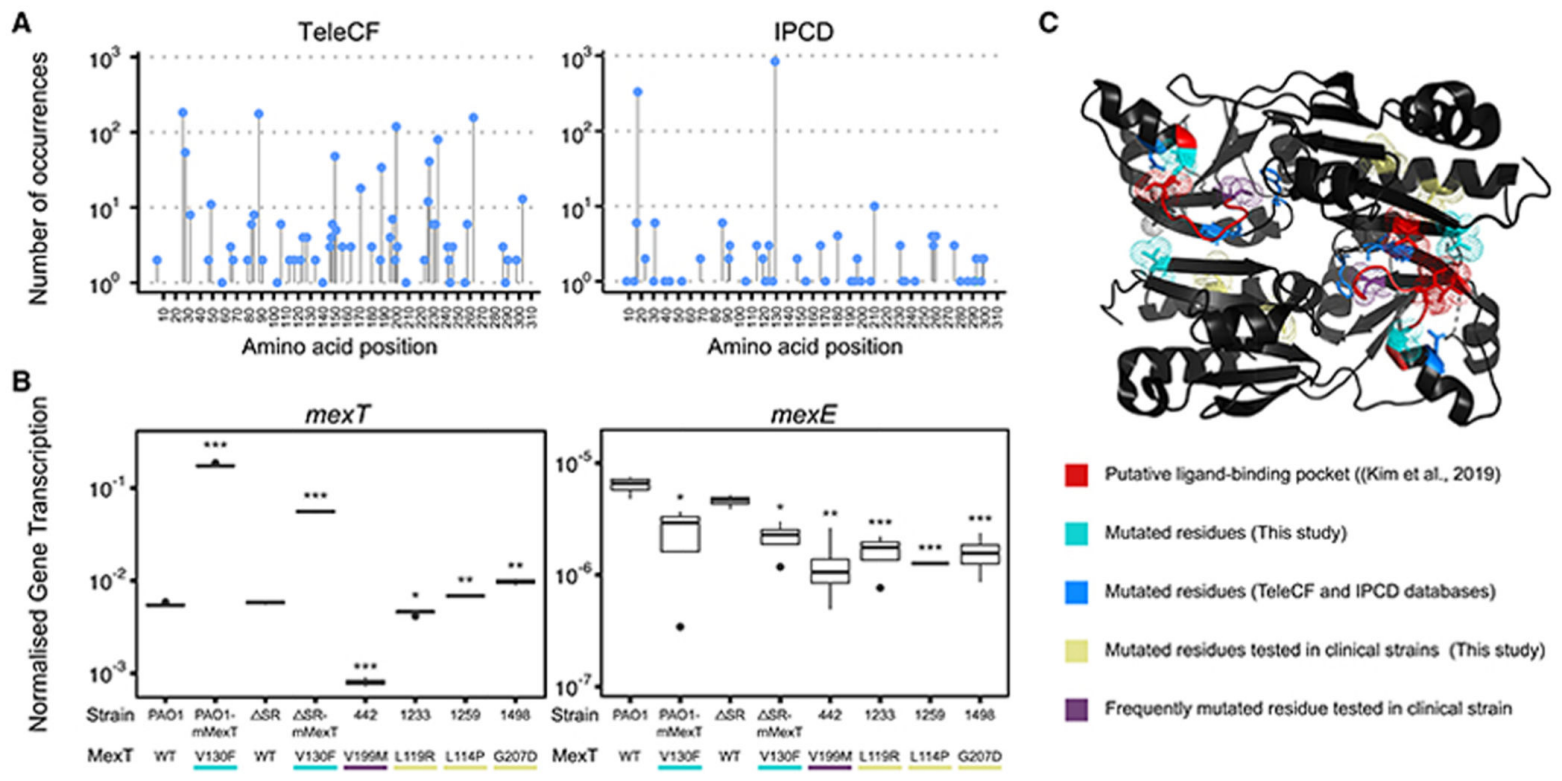
Prevalence and distribution of *mexT* mutations in diverse *P. aeruginosa* strains The presence of mutations in *mexT* was assessed by variant calling analysis of the genome sequences of *P. aeruginosa* isolates harvested for an in-house study (TeleCF) and from the publicly available IPCD collection. (A) Graphs showing the frequency of non-synonymous SNPs in the TeleCF and IPCD database, as indicated, and the position of the amino acid affected in the linear sequence of the protein (Data S6). Blue dots indicate missense mutations; green dots denote frameshift mutations. (B) RT-qPCR of a selection of clinical or environmental IPCD strains was performed for the *mexT* and *mexE* ORFs and is represented as transcription normalized to the abundance of the housekeeping gene, *rpoD* (*n* = 3). (C) Residues most commonly affected by NS-SNPs (>5 occurrences) were mapped onto the X-ray crystal structure of the MexT ligand-binding domain (PDB: 6l33) and are shown in blue. Residues comprising the putative ligand-binding site in the domain are shown in red. The two residues (V130 and G207) identified in our experimental screen are shown in cyan. Residues mutated in the IPCD strains tested by RT-qPCR are shown in yellow. Where the most frequently mutated residues coincide with the tested clinical strains, the amino acid is shown in purple. Where they are used, the asterisks denote statistically significant differences compared to PAO1 based on Welch’s t test, using a Bonferroni correction (*p < 0.05, **p < 0.01, ***p < 0.001).

Our data indicate that mutations in *mexT* can “re-wire” the *P. aeruginosa* transcriptome and have an extensive impact on the biology of the organism, in particular, with regard to virulence and antimicrobial resistance. Importantly, *mexT* has been high-lighted in recent studies as a hotspot for mutation in the laboratory strain, PAO1.^17^ This prompted us to investigate the distribution of *mexT* mutations across a wide variety of *P. aeruginosa* strains. We first looked at the *mexT* sequences in an in-house collection of strains derived from the airways of people with CF. These strains were isolated over a period of 6 months from 15 adults with CF (part of the Telemed study [TeleCF]).^18^ Non-synonymous single-nucleotide polymorphisms (NS-SNPs) were identified relative to the PAO1 reference sequence. We found that mutations in *mexT* were strikingly common, with some residues (e.g., residue 17 in the DNA-binding domain, and residues 199 and 262 in the ligand-binding domain) mutated in >100 isolates (~10% of all TeleCF isolates) (Figure 4A).

We next studied the prevalence of *mexT* mutations in the International *Pseudomonas* Consortium Database (IPCD), a collection of 854 isolates obtained from diverse environments (e.g., the CF airways, urinary tract infections, wounds, burns, keratitis, plants, rivers, soil, and animals) in 13 different countries^19^ (Data S6). Most of the mutations identified in the IPCD collection were missense mutations, although some frameshift variants were also detected (Figure 4A). Interestingly, although we identified many NS-SNPs in *mexT* among the IPCD isolates, the frequency of these SNPs was lower than it was in isolates from the Telemed study. We wondered whether this might reflect the original source of the strains in the IPCD, e.g., environmental vs. clinical, or different wound types etc. We therefore re-interrogated the data based on the origin of each isolate. Mutations in *mexT* were detected in *P. aeruginosa* from all of the sources identified (Figure S5). In particular, CF isolates displayed a higher frequency and diversity of *mexT* mutations (Figure S5). Although it is worth noting that CF-associated isolates account for more than half of the total genomes in the IPCD database (Figure S5), a similarly high frequency of *mexT* mutation was seen in the TeleCF dataset (along with other genes associated with CF adaptation^18^), suggestive of host-specific adaptation.

We selected some clinical and environmental isolates from the IPCD collection (Table S4) carrying mutations near the putative ligand-binding site (including the frequently mutated residue, V199, and the residue mutated in this study, G207) and used quantitative reverse-transcription PCR (RT-qPCR) to monitor the transcript abundances of *mexT* and *mexE* as indicators of MexT function (Data S7). Control strains (PAO1-mMexT and ΔSR-mMexT) showed the same expression patterns as the RNA-seq data when compared against PAO1; i.e., *mexT* was upregulated and *mexE* downregulated (Figure 4B). However, the abundance of *mexE* transcript in the IPCD strains was the same or even lower than that the control strains (Figure 4B), suggesting that the *mexT* mutations present in those isolates do indeed lead to loss of function.

The *mexT* mutations identified in the TeleCF and IPCD genome collections showed no obvious pattern of clustering in the protein primary structure (Figure 4A). However, when we mapped the affected amino acids onto the recently published X-ray crystal structure of the MexT ligand-binding domain,^10^ the most frequently affected residues in clinical CF isolates (including the ones we tested by qPCR), and residues V130 and G207, were all located in or near to the putative ligand-binding pocket (Figure 4C). Interestingly in this regard, one of the TeleCF patients was found to be infected by a *P. aeruginosa* strain carrying the G207S substitution in MexT. Inspection of the accompanying clinical metadata for this patient revealed that the patient was being treated with nebulized colistin, nebulized meropenem, and intravenous tobramycin. Taken together, our results indicate that *mexT* mutations are widespread in the clinic and that these mutations are enriched in the vicinity of the ligand-binding pocket of the protein. Furthermore, it seems possible that such mutations might even be selected for by the use of certain antibiotics in the CF airway environment.

### Mutations in *mexT* are associated with elevated lethality and bacteremia in a mouse model of pulmonary infection

**Figure 5:**
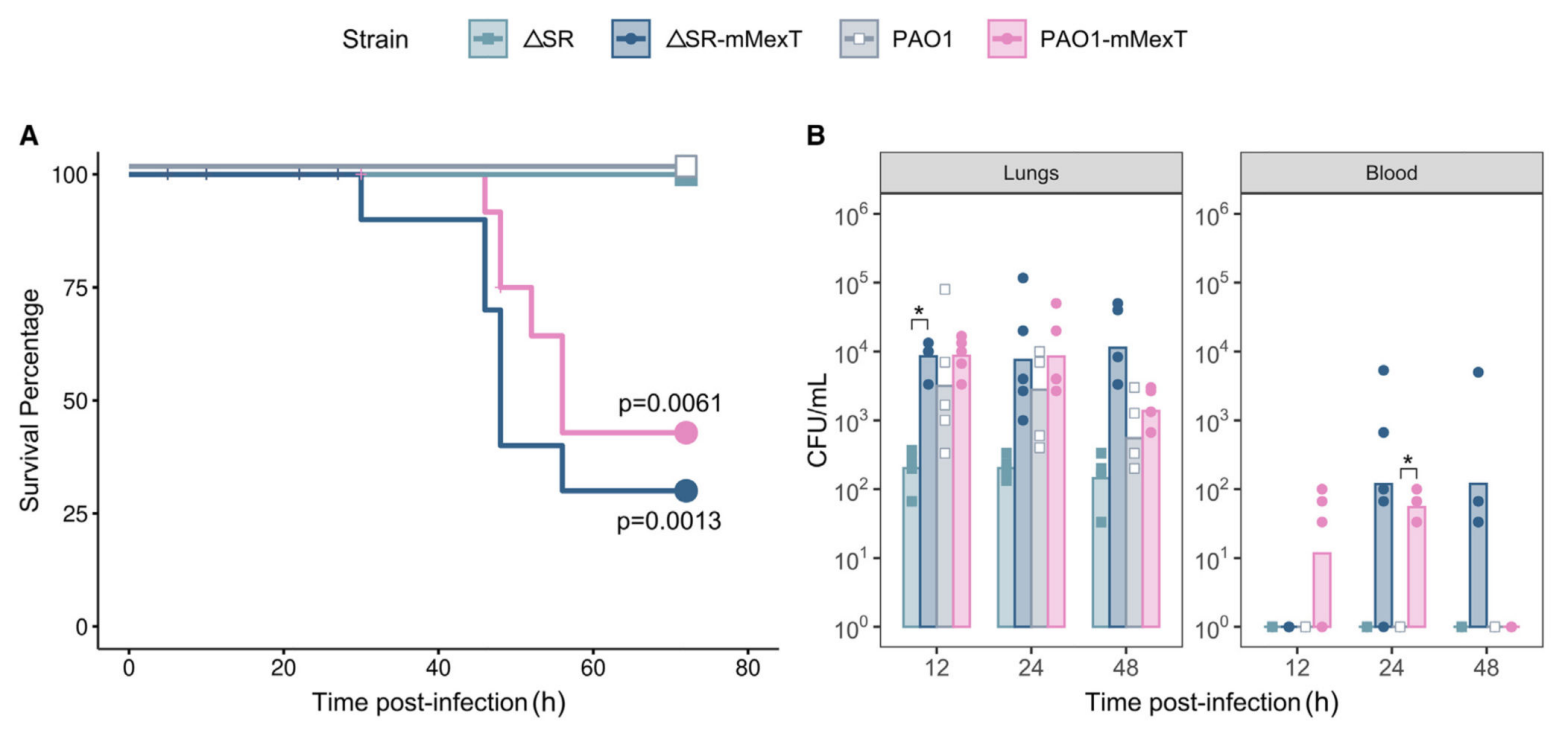
*MexT* mutants are hypervirulent in a mouse pulmonary infection model (A) Kaplan-Meier curve showing the survival rate over 72 h of mice intranasally challenged with 5 × 10^4^ CFU of PAO1, ΔSR, PAO1-mMexT, or the ΔSR-mMexT strain. Mice were monitored for signs of ill health and were culled when the severity threshold was reached. Results are a combination of two independent experiments, with *n* = 10 mice per group. The *p* value shown was determined using the Mantel-Cox test comparing PAO1-mMexT or ΔSR-mMexT with their respective parental strains. (B) Bacterial CFUs in the lungs and blood following intranasal challenge (*n* = 5 mice per group). Data are represented as bars indicating the mean and the individual replicates as dots. The asterisks denote statistically significant differences determined via Welch’s t test comparing PAO1-mMexT or ΔSR-mMexT with their respective parental strains (*p < 0.05).

SR-null *P. aeruginosa* mutants have been shown to have a significantly reduced ability to establish infections in *Galleria mellonella, Caenorhabditis elegans*, and murine models.^20^ Given that our findings demonstrate that SR deficiency can be bypassed by the loss of MexT, leading to the upregulation of genes related to virulence factor production and bacterial survival in the host, we next investigated the impact of such mutations *in vivo*. Since we found that mutations in this regulator are prevalent across CF isolates, we used a mouse model of pulmonary infection. The mice were intranasally challenged with either PAO1, the ΔSR mutant, PAO1-mMexT, or the ΔSR-mMexT strain. All mice challenged with the parental strains (PAO1 and ΔSR) survived the full 72-h time course. By contrast, the survival rate was significantly lower for mice challenged with PAO1-mMexT (survival percentage 42%, *p* < 0.0061) or ΔSR-mMexT (survival percentage 32%, *p* < 0.0013) when compared with their respective parental strains (Figure 5A; Data S8). These data indicate that the absence of a functional MexT in either the ΔSR or wild-type backgrounds leads to a hypervirulent phenotype *in vivo*. To further investigate this, we measured *P. aeruginosa* titers (colony-forming units [CFU]) in the lungs and blood of the mice 12, 24, and 48 h post challenge. The bacterial load in the lungs of mice challenged with PAO1 and PAO1-mMexT was similar throughout the 48-h time course. However, the bacterial load of mice challenged with ΔSR-mMexT (≤10^5^ CFU/lung) was significantly higher (*p* = 0.0005) than that of the parental ΔSR strain (≤10^3^ CFU/lung) at all measured time points and was comparable throughout to bacterial loads seen in the SR-proficient strains, PAO1 and PAO1-mMexT (Figure 5B; Data S9). The *mexT*^V130F^ allele was also associated with bacteremia; although no bacteria could be cultured from the blood of mice challenged with the parental strains PAO1 and ΔSR, bacteremia was observed in the mice challenged with PAO1-mMexT and with ΔSR-mMexT (Figure 5B, Data S10). The enhanced dissemination of strains containing the *mexT*^V130F^ allele from the lungs into the bloodstream presumably contributes to the decreased survival of mice challenged with these strains. Importantly, the ΔSR-mMexT strain also appeared to persist for longer than PAO1-mMexT in the bloodstream, which may suggest subtly different mechanisms of invasion.

In summary, our data reveal that the SR is not essential for virulence in *P. aeruginosa* and that the mutational status of *mexT* is even more important in the hierarchical structure that controls virulence. Our data show that some mutations in *mexT* not only activate virulence independent of the SR; they also lead to elevated resistance to clinically important antibiotics—a combination of phenotypes that is both unusual and unwelcome.

## Discussion

Many pathogens employ cell-cell communication via QS as a means of regulating virulence. Perhaps less well appreciated is the fact that they only produce virulence factors—even when the population is quorate—when they are short of nutrients and that nutrient availability is titrated by the SR.^20^ An example of how bacteria can adapt to overcome the absence of (p)ppGpp was reported by Bowden et al.^21^ In their study, they showed that *Pectobacterium atrosepticum* can bypass the SR via a disruption of the gene *rsmA* to restore the production of plant cell-wall-degrading exoenzymes.^21^ By contrast, our data demonstrate that *P. aeruginosa* employs a very different mechanism, involving *mexT*, to bypass the SR for virulence factor production.

*MexT* encodes a LysR-type transcriptional regulator (LTTR) that is thought to be regulated by the binding of an unknown ligand. MexT positively stimulates expression of the *mexEF*-*oprN* efflux pump.^22^ Besides the *mexEF-oprN* efflux pump, several genes have been identified to be directly regulated by MexT in *P. aeruginosa*, such as the porin *oprD*, the *pqs* biosynthetic operon, and genes involved in hydrogen cyanide synthesis. This highlights the role of MexT as a global transcriptional regulator.^23^ Interestingly, in our RNA-seq experiment, we found that, in the wild-type background (PAO1), the main genes upregulated in the *mexT*^V130F^ mutant were associated with the T6SS (almost half of all upregulated genes), transcriptional regulators (*mexT* and *nalC*), and the *mexGHI-opmD* efflux pump. On the other hand, most of the downregulated genes (in addition to the *mexEF-oprN* efflux pump) were of unknown function.

The MexEF-OprN pump exports ciprofloxacin but shows no activity against aminoglycosides.^12^ It was therefore surprising to see that the restoration of virulence associated with *mexT* mutants was also accompanied by resistance to aminoglycoside antibiotics and to the last-resort antibiotic, colistin. It has been reported that aminoglycosides interact with the LPS layer on gram-negative bacteria.^24^ This is also a well-known target of colistin.^25^ Consistent with this, modifications of the LPS layer have been associated with resistance to aminoglycosides and colistin.^25–27^ Furthermore, we have previously reported that loss-of-function mutations in genes involved in the O-antigen biosynthesis pathway can confer resistance to colistin. In this regard, we note that transcripts associated with O-antigen biosynthesis are downregulated in the *mexT* mutants, suggesting that this could be the mechanism for resistance against both colistin and aminoglycosides.

Some previous studies have independently established that mutations in *mexT* enable the restoration of exo-protease production in *lasR* (QS defective) mutants of *P. aeruginosa*,^28,29^ suggesting that QS-associated signals may be routed through MexT. LasR is a LuxR-type regulator at the top of the QS hierarchy in the organism, and *lasR* mutants are almost entirely avirulent because they fail to produce OdDHL and, as a consequence, also fail to produce BHL. However, BHL synthesis is restored in *lasR mexT* mutants, even in the absence of OdDHL.^28,29^ Interestingly, synthesis of *both* OdDHL and BHL is abolished in a ΔSR mutant,^6^ yet our data demonstrate that the synthesis of both molecules is restored to wild-type levels in the ΔSR-mMexT bypass strain. These data indicate that MexT is positioned high up in the regulatory hierarchy controlling virulence factor production.

In addition to exporting quinolone antibiotics, MexEF-OprN also exports 4-hydroxy-2-heptylquinoline (HHQ), which is a water-soluble bioactive intermediate in the production of PQS.^30^ Consequently, the loss of *mexEF*-*oprN* expression in a *mexT* mutant may lead to an accumulation of intracellular HHQ (and therefore also PQS). This, in turn, should elicit activation of the *rhl* system.^31^ Intriguingly, a second resistance-nodulation-division (RND)-family multidrug efflux pump, *mexGHI-opmD*, was also upregulated (8-fold) in the PAO1-mMexT strain. This pump has also been previously linked with virulence in *P. aeruginosa*; Aendekerk et al. found that null mutations in either *mexI* or *opmD* resulted in an inability to produce OdDHL and PQS, and that BHL concentrations are greatly lowered,^32^ thus leading to the loss of virulence. Elevated expression of the pump would be expected to reverse these traits. Although the concentration of QS molecules, and *lasR, rhlR*, and *pqsR* transcripts, were essentially unaffected in PAO1-mMexT, we note that the production of virulence factors such as pyocyanin (observed as a blue pigment) and proteases started earlier in the growth curve compared with the progenitor PAO1.

Regarding the *in vivo* hypervirulent phenotype, we note that the H2-T6SS (type VI secretion system) was 8-fold upregulated (on average) in both the PAO1-mMexT and the ΔSR-mMexT strains, accounting in the former for nearly half of all the upregulated genes (11 out of 25). Type VI secretion systems have been reported to intoxicate both prokaryotic and eukaryotic cells. In particular, the H2-T6SS, whose regulation is still not completely understood, has recently been linked to internalization into eukaryotic cells via a PI3K/Akt-dependent pathway.^33,34^ One of the H2-T6SS effectors is PldA, a phospholipase D associated with the killing of bacterial competitors and internalization into non-phagocytic cells.^35^ The *pldA* gene is not universally present in all *P. aeruginosa* isolates, and a recent study uncovered a high prevalence of *pldA* in *P. aeruginosa* isolates associated with severe acute pulmonary infection and septicemia. *PldA* was ca. 2.5-fold upregulated in PAO1-mMexT and nearly 9-fold upregulated in ΔSR-mMexT. These data suggest that the hypervirulent phenotype of *mexT* mutants *in vivo* might be at least partially attributable to high levels of T6SS-dependent PldA secretion.

The mouse pulmonary infection model also revealed another unexpected finding, that blood CFUs were not simply a reflection of the lung CFU load. PAO1 and PAO1-mMexT had almost the same lung CFU load throughout the time course examined, yet only PAO1-mMexT led to sepsis. Similarly, the ΔSR-mMexT strain (which had the highest lung tissue titers of any of the strains tested) also caused sepsis, whereas its immediate progenitor (ΔSR) did not. In this regard, we found that genes such as *loxA* (which has been found to modulate the host immune response and increase bacterial survival in lung tissue in a mouse model^36^), and *cbpD* (which enhances survival in human blood^37^) were highly upregulated in PAO1-mMexT and ΔSR-mMexT, respectively. Taken together, our data suggest that (1) sepsis is relatively unrelated to lung titers perse, and (2) that the *mexT*^V130F^ mutation likely elicits its virulence effects via different mechanisms in the wild-type and ΔSR backgrounds.

Intriguingly, one of the most upregulated genes in both the ΔSR-mMexT and PAO1-mMexT strains was *mexT* itself (12- and 20-fold, respectively). Thus far, the mechanisms regulating *mexT* expression remain not clearly understood. Some authors propose that MexT activity/expression is regulated by a redox mechanism that also involves *mexS* (which encodes an oxidoreductase). This is because mutations in *mexS* are linked with overexpression of the MexT-dependent *mexEF-oprN* efflux pump.^38,39^ Additionally, Jin et al. provided evidence of a weak interaction between MexS and MexT,^40^ possibly suggesting that MexT may autoregulate. Unfortunately, it remains to be elucidated whether MexS is solely responsible for the regulation of *mexT* expression or whether more players remain to be found.

Our mutational analyses show that *mexT* is a mutational hotspot preferentially associated with CF isolates. This is interesting since CF-associated strains are usually considered to be less virulent. However, here we show that one of the most frequently mutated residues in the TeleCF collection (V199) seems to lead to loss of function (based on our RT-qPCR data), thus raising the question of whether CF isolates really do produce less virulence factors. We hypothesize that strains in acute infections produce virulence factors to thrive, whereas CF isolates linked to chronic infections may produce them to survive.

Our results show that the functional status of MexT exerts a crucial influence on the physiology of the cell, acting as a “genetic switch” depending on the mutations it harbors. It is well known that some activating mutations in this regulator (ON state) lead to the overexpression of the MexEF-OprN efflux pump, low virulence, and concomitant resistance to fluoroquinolones.^22,41^ Here we uncovered a class of mutations that, through inactivation of the regulator (OFF state), supersede the SR, leading to hypervirulence and resistance to aminoglycosides and the last-resort antibiotic colistin. This positions *mexT* as a key player in the evolution and adaptation of *P. aeruginosa* since its mutational state influences a larger and more intricate regulatory genetic circuit. In this scenario, different mutations in the same gene (*mexT*) can potentially lead to very different outcomes. Consistent with this notion, our data show that *mexT* is commonly targeted by mutations in *P. aeruginosa* clinical isolates, implying that antibiotic use in the clinic may play a role in selecting for these.

### Limitations of the study

The current study experimentally tested the effect of only a subset of mutations commonly occurring in *mexT*. Although most clinical *mexT* mutations appear to cluster around the probable ligand-binding site in the protein (as is also the case for the V130F and G207S mutants studied here), it is possible that other *mexT* mutations may have a different impact on phenotype. We also note that, currently, we can only speculate on the molecular mechanism(s) linking loss-of-function *mexT* mutations with the restoration of QS and virulence, and elucidation of the pathways involved remains a key future goal.

## Resource Availability

### Lead contact

Further information and requests for resources should be directed to the lead contact, Martin Welch (mw240@cam.ac.uk).

### Materials availability

Strains and plasmids generated in this study are available from the lead contact upon request.

## Star★Methods

### Key Resources Table

**Table:** 

REAGENT or RESOURCE	SOURCE	IDENTIFIER
Bacterial and virus strains		
PAO1 wild-type	Dao Nguyen^42^	N/A
PAO1 ΔrelA ΔspoT(ΔSR)	Dao Nguyen^42^	N/A
JM109	Michael Winson [S4]	N/A
JM109	Michael Winson [S4]	N/A
PAO1 ΔpqsA CTX-lux::pqsA	Paul Williams [S5]	N/A
ΔSR-mMexT	This paper	N/A
PAO1-mMexT	This paper	N/A
PAO1-ΔMexT	This paper	N/A
Critical commercial assays		
RNAlater	Invitrogen	Cat# AM7024
RNeasy Kit	Qiagen	Cat# 74104
SuperScript IV VILO Master Mix with ezDNase Enzyme	Invitrogen	Cat# 11766050
PowerUp™ SYBR™ Green Master Mix for qPCR	Applied Biosystems	Cat# A25742
Deposited data		
RNA-seq data	This paper	GenBank: PRJNA94747
IPCD genomes	Freschi et al.^19^	GenBank: PRJNA325248
TeleCF genomes	Weimann et al.^18^	ENA: ERP022089
Experimental models: Organisms/strains		
BALB/c mice	Harlan Laboratories	BALB/cOlaHsd
Software and algorithms		
Breseq	Deatherage et al.^43^	https://github.com/barricklab/breseq
Fastqc	Andrews S.^44^	https://github.com/s-andrews/FastQC
BWA	Li et al.^45^	https://github.com/lh3/bwa
Samtools	Li et al.^9^	http://samtools.sourceforge.net/
FeatureCounts	Liao et al.^46^	https://doi.org/10.1093/bioinformatics/btt656
Cytoscape	Shannon et al.^47^	https://cytoscape.org
StringApp	Doncheva et al.^48^	https://apps.cytoscape.org/apps/stringapp
Omics Visualizer app	Legeay et al.^49^	https://apps.cytoscape.org/apps/omicsvisualizer

### Experimental Model And Study Participant Details

For all *in vivo* experiments, 6–8-week-old female BALB/c mice were used. The mice were allowed to acclimatise for 7 days prior to use under conditions described previously.^50^ All experimental protocols were approved and performed in accordance with the regulations of the Home Office Scientific Procedures Act (1986), project license P86De83DA and the University of Liverpool Ethical and Animal Welfare Committee.

## Method Details

**Phenotypic assays** (biofilm formation, motility, siderophore production, exoprotease activity, pyocyanin production, quorum sensing molecule bioassay, antibiotic susceptibility and MIC determination) are described in the Methods S1 section.

### Bacterial strains and culture conditions

The PAO1 wild-type (PAO1) and the stringent response mutant (PAO1 Δ*relA* Δ*spoT* or ΔSR) were kindly provided by Dr Dao Nguyen from McGill University^42^ (Table S4). Overnight cultures were grown routinely in lysogeny broth (LB-Lennox, Oxoid) with shaking at 37°C unless otherwise indicated. In the case of the assays that measure virulence factors, 50 mL cultures were grown in 250 mL flasks with agitation at 200 rpm.

### Isolation of Gm^R^ Prt^+^ bypass mutants

The ΔSR mutant was deficient in the production of secreted protease. We initially set out to identify gentamicin-resistant bypass mutants in which secreted protease production was restored following a transposon mutagenesis campaign. Briefly, the recipient strain (ΔSR), the *E. coli* host containing the plasmid-encoded transposon (pTnMod-OGm), and *E. coli* HB101 containing the helper plasmid pRK2013 were grown in LB to an OD_600_~0.7–1.0. Aliquots (1 mL) of each strain were combined and sedimented at 2,348 × *g* for 5 min. The supernatant was discarded, and the cell pellet was resuspended in 50 µL of LB. The cell suspension was then plated onto 40 non-selective LB-agar (1.6% agar) plates and incubated for 16 h at 37°C. The colonies recovered from the plates were resuspended in 1 mL of 10% (v/v) glycerol, and 100 μL of each mating solution were plated onto skim-milk agar plates containing 50 µg/mL gentamicin and 20 μg/mL chloramphenicol (to counter-select the *E. coli*). The plates were incubated for 2 to 3 days. Around 120,000 individual Gm^R^ colonies were assessed and selected based on their ability to produce secreted proteases (assessed by the presence of a clear halo of hydrolyzed milk protein around each colony). No transposon was detected in the bypass (TM) mutants by PCR so a selection of the Gm^R^ Prt^+^ isolates were subjected to whole genome sequencing.

### Sequencing of bypass mutants and variant calling

Genomic DNA from selected TM strains was extracted following a previously described protocol.^51^ Birefly, 1.5 mL of an overnight culture was pelleted and resuspended in 200 µL of lysis buffer (40 mM Tris-acetate pH 7.8, 20 mM sodium-acetate, 1 mM EDTA, 1% SDS). Proteins and cell debris were removed by adding 66 µL of 5M NaCl, followed by centrifugation at 12,000 rpm for 10 min at 4°C. The clear supernatant was transferred to a new tube, an equal volume of chloroform was added, and mixed by inversion followed by centrifugating at 12,000 rpm for 3 min. The extracted supernatant was transferred to another tube and the DNA was precipitated with absolute EtOH, and washed twice with 70% EtOH. The resulting pellet was resuspended in 50μL of water. The DNA samples were submitted for whole-genome sequencing (carried out by MicrobesNG using an Illumina HiSeq 2500 platform, and 2 x 250 bp paired-end reads). Variants were called using Snippy v2.5 (https://github.com/tseemann/snippy) and Breseq v0.35.0^43^ using the default settings. Mutations were identified using PAO1 (NC_002516) as reference and were compared with the ones already present in the parental strain (ΔSR) to identify unique mutations. Called variants were visually inspected by mapping the reads on the reference genome using Artemis.

### Genome engineering

The reconstruction of individual mutations was performed by allelic exchange following the protocol adapted for *Pseudomonas aeruginosa* by Hmelo et al.^52^ Briefly, the segment of DNA containing the missense allele of *mexT* (V130F) was PCR-amplified using the genomic DNA from mutant TM-62 as template. The resulting amplified DNA was cloned in the plasmid pEX19Gm. In parallel, and to generate a *mexT* deletion mutant, two PCR amplifications were carried out; one to amplify the flanking regions of the *mexT* (~500 bp on each side), and one to join both fragments into a single entity. The resulting DNA fragment was also cloned into pEX19Gm. Both plasmid constructions (pEX19Gm-mMexT and pEX19Gm-ΔMexT) were introduced into *Pseudomonas* by electroporation following the protocol established by Choi et al.^53^ Merodiploids were selected on LB-Gm^80^ plates. Positive candidates were transferred to a second LB plate containing 15% sucrose for counter-selection against the merodiploids. Around 10 colonies from each plate were tested by PCR to confirm the deletion, or by Sanger sequencing to detect the missense mutation.

### MexT complementation

*MexT* wild-type and mutant alleles were cloned (separately) into the KpnI and BamHI sites of pUCP20. Plasmid constructions pUCP20(*mexT*)wt and pUCP20(*mexT*)mut were introduced into strains ΔSR, ΔSR-mMexT, PAO1 WT, PAO1-mMexT, and PAO1Δ*mexT* by electroporation, and plated onto LB-Cb^300.52^

### Sample preparation for RNA-seq

Overnight cultures of three biological replicates of the different mutants were diluted 1:500 in fresh LB-MOPS medium and incubated at 37°C and with good aeration (shaking at 200 rpm). After 8 h of growth, 15 mL of culture were pelleted (2348 × *g*) and washed with 10 mL of RNAlater (Invitrogen, Catalog number: AM7024). The bacterial suspensions were then pelleted again (2348 × *g*) for 5 min, and the pellets were resuspended in 1 mL of RNAlater. Samples were stored at -20°C and shipped on dry ice to Eurofins-Germany for RNA extraction, rRNA depletion, and sequencing.

### RNA-seq analysis

The raw RNA-seq data was first assessed for quality using fastqc v0.11.5.^44^ The reads were then aligned to the reference PAO1 genome (NC_002516) using BWA-MEM,^45^ and the quality of the alignment was assessed using samtools flagstat v1.8.^54^ Reads mapping to the coding regions of the genome were counted with featureCounts v1.5.0-p1,^46^ and a Principal Component(s) Analysis (PCA) was carried out with the output data to visualise global differences in the expression profile of each sample (*n* = 3 replicates). Finally, a differential expression analysis was performed in R 3.6.0 using the DESeq2 package. Data has been deposited under the accession number GenBank: PRJNA947473.

### Network analysis

Functional interactions among the 588 genes/ORFs identified as differentially expressed in the ΔSR-mMexT vs. ΔSR mutants were explored via a network analysis using the STRING database and Cytoscape v3.8.1.^47^ Functional interactions were retrieved from STRING using the stringApp v1.7.1^48^ with an input list of locus tags for the 588 genes/ORFs, selecting “*Pseudomonas aeruginosa*” as the target species, “0.7” as the confidence score (high confidence), and otherwise default settings. Out of the 588 input locus tags, functional interaction data was retrieved for 532 proteins; the remaining did not map an STRING identifier under the parameters given. The retrieved network was visualised by applying the “Edge-weighted Spring Embedded” layout with the attribute score. A functional enrichment analysis for the proteins in the network was performed with the “Functional Enrichment” option from the “STRING enrichment” app and using the *Pseudomonas aeruginosa* whole-genome network as background. The resulting table indicating the enriched terms, corresponding FDR values, and information for each enrichment category, was used to annotate the network (Figure 4). The network topology metrics were obtained using the Cytoscape v3.8.1 “Analyze Network” function with the default parameters. (i.e., undirected network). Central genes were determined based on their closeness, radiality and eccentricity values. Finally, RNA-seq differential expression data were mapped to the proteins and overlaid on the network using the Omics Visualizer app.^49^ Log_2_ fold-change (L2FC) values for both the “PAO1-mMexT vs. PAO1” and “ΔSR-mMexT vs. ΔSR” comparisons were mapped as different rings surrounding the nodes in the network to compare the direction and magnitude of the genes’ expression in the two different genomic backgrounds studied here.

### Analysis of diversity and prevalence of mutations in *mexT* clinical and environmental isolates

The International *Pseudomonas* Consortium Database (IPCD), comprised of 854 *Pseudomonas aeruginosa* genomes, was downloaded from GenBank: PRJNA325248. Variants for all the genomes were called and extracted from the variant calling files using the term “*mexT*”. The output data were compiled in a table. The metadata from the genome sequence files was extracted from the IPCD website (https://ipcd.ibis.ulaval.ca) and added to the mutations table. Analysis and visualisation of mutations in *mexT* were performed in R 3.4.0. Similar analyses were carried out for the TeleCF database (ENA: ERP022089).

### Quantification of gene expression by RT-qPCR

A selection of IPCD and control strains were grown in the same conditions as those used for the RNA-seq sample preparation. RNA was extracted using the Qiagen RNeasy Kit (Catalog number 74104) according to the manufacturer’s instructions. Reverse transcription was performed using SuperScript IV VILO Master Mix with ezDNase Enzyme (Catalog number 11766050). Quantitative PCR was then used to quantify cDNA encoding the *rpoD, mexT* and *mexE* transcripts. The qPCR was carried out on *N* = 4 independent sample replicates using PowerUp SYBR Green Master Mix for qPCR (Catalog number A25742) in an applied biosystems QuantStudio 6 Flex instrument. Expression of *mexT* and *mexE* was normalised to that of *rpoD*.

### Mouse pulmonary infection model

Mice were anesthetized with a mixture of O_2_ and isoflurane and challenged intranasally with 5 × 10^4^ CFU of either PAO1, PAO1-mMexT, ΔSR, or ΔSR-mMexT.^55^ For the survival experiment, mice were monitored regularly for signs of ill health and were culled before the severity threshold was reached or at the designated end point (72 h).^46^ For the time-course experiment, mice were culled as described previously,^50,56^ and the lungs and blood were dissected and homogenised. The bacterial load was then established via colony-forming units (CFU) on *P. aeruginosa* selective plates.

## Quantification And Statistical Analysis

All the statistical details of experiments can be found in the figure legends. Significance was defined as any adjusted p-value <0.05.

## Supplementary Material

Supplemental information can be found online at https://doi.org/10.1016/j.celrep.2024.115079.

Supplementary Materials

## Data Availability

Data: RNA-seq data have been deposited in GenBank: PRJNA94747. All genomes used in this study can be found under the accession numbers GenBank: PRJNA325248 (IPCD genomes) and ENA: ERP022089 (TeleCF database). Code: this paper does not report original code. Other items: any other resource not stipulated here can be requested from the lead contact.

## Highlights

- Virulence can be activated independently of the stringent response via mutations in *mexT*
- Loss of MexT drives enhanced host colonization, hypervirulence, and multidrug resistance
- MexT acts as a genetic switch, toggling the virulence state of *P. aeruginosa* via mutations

## In brief

Regulatory systems behind antimicrobial resistance and virulence are poorly understood. Figueroa et al. show that the mutational state of the *Pseudomonas aeruginosa* regulator *mexT* acts as a genetic switch, controlling virulence and antibiotic resistance. They further show that *mexT* mutants are common in clinical isolates and exhibit hypervirulence *in vivo*.
