# Supplementary material for: Mutations in mexT bypass the stringent response dependency of virulence in Pseudomonas aeruginosa
Source: Cell Rep. Author manuscript; Available in PMC 2025 Nov 18. (PMC7618369; doi:10.1016/j.celrep.2024.115079)
Supplement: Supplementary Materials [file EMS209706-supplement-Supplementary_Materials.zip › 1-s2.0-S221112472401430X-mmc1.pdf]

Cell Reports, Volume *44*

Supplemental information

Mutations in *mexT* bypass the stringent

response dependency of virulence

in *Pseudomonas aeruginosa*

Wendy Figueroa, Adrian Cazares, Eleri A. Ashworth, Aaron Weimann, Aras Kadioglu, R. Andres Floto, and Martin Welch

Supplementary Information

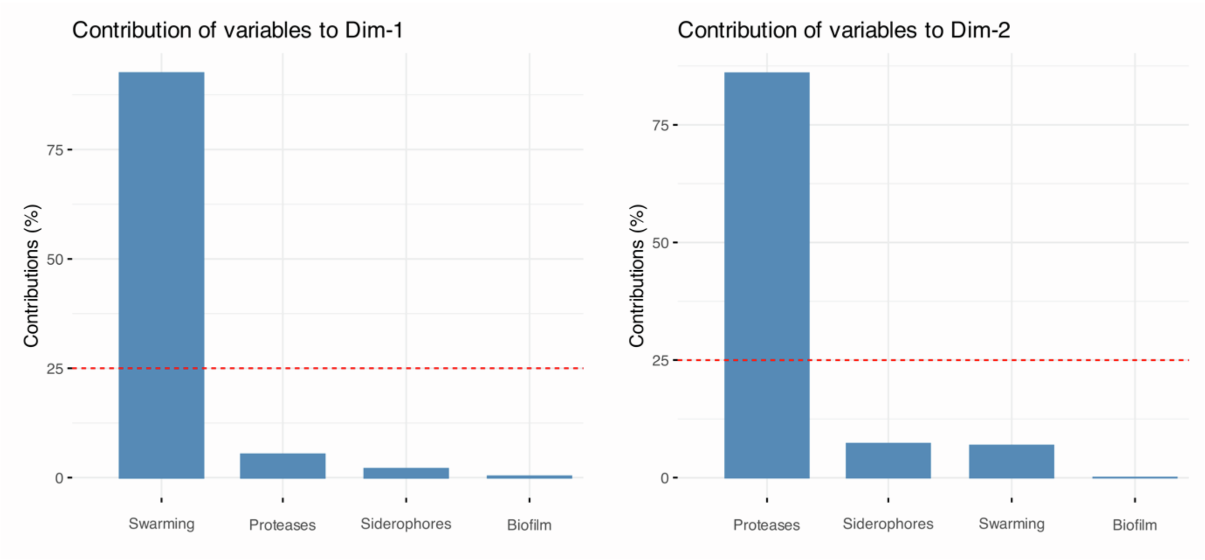

Figure S1. Variables contribution to PCA, Related to Figure 1. Contribution of each variable used in the Principal Component(s) Analysis (PCA) to dimension 1 (left panel) and dimension 2 (right panel).

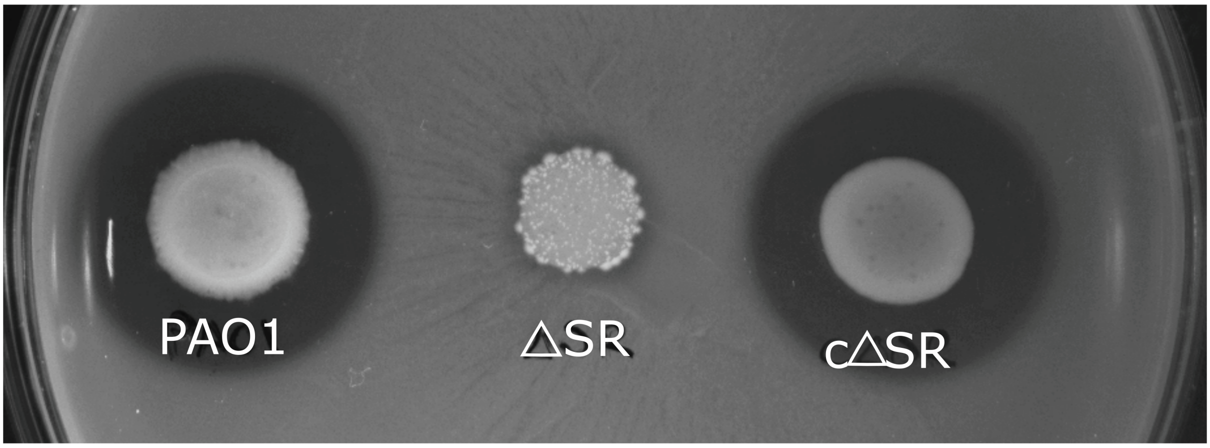

Figure S2. Bulk proteases assay on a plate, Related to Figure 1. Production of exo-proteases by PAO1 wild-type strain, stringent response mutant (Δ*relA* Δ*spoT*) and Δ*relA* Δ*spoT* mutant complemented with the *relA* and *spoT* genes (introduced at a neutral site in the chromosome) on agar plates containing skimmed milk. Protease production is measured as the diameter of the clear halo that surrounds the bacterial colonies/spots.

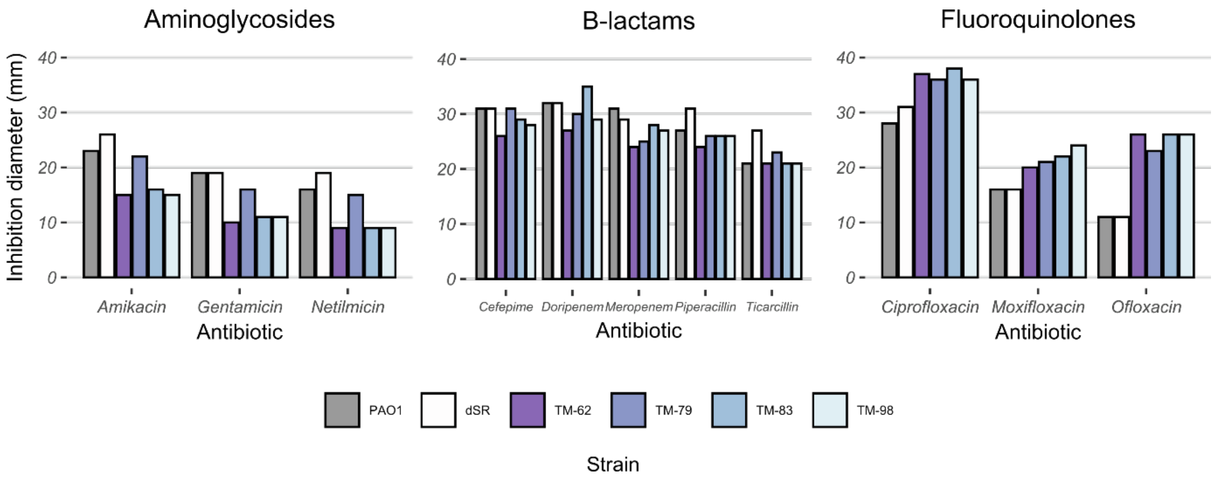

Figure S3. Antimicrobial susceptibility test on discs, Related to Figure 2. The figure shows the inhibition diameter of different antibiotics belonging to the aminoglycoside, β-lactam or fluoroquinolone classes for the bypass mutants. Results from this assay were used to select representative antibiotics for further analyses.

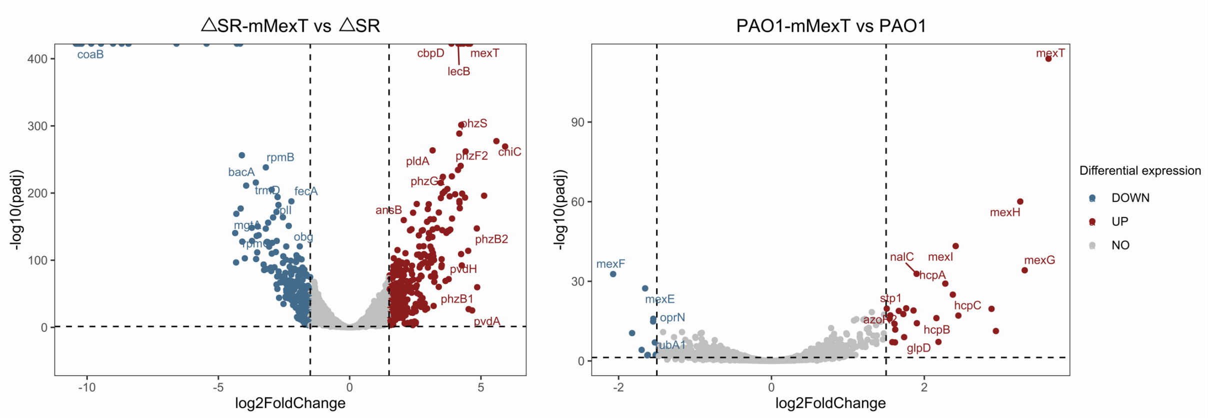

Figure S4. Differential expression analysis of RNA-seq data, Related to Figure 3. The figure shows volcano plots where the $\log_2$ fold-change is shown on the *x*-axis, and the $-\log_{10}$ adjusted p-value on the *y*-axis. Dotted lines represent the cut-off values used in the analysis (±1.5 $\log_2$ fold-change, and adjusted p-value <0.05). Dots in grey indicate no differential expression, whereas dots in blue or red indicate that the genes were down-regulated or up-regulated, respectively.

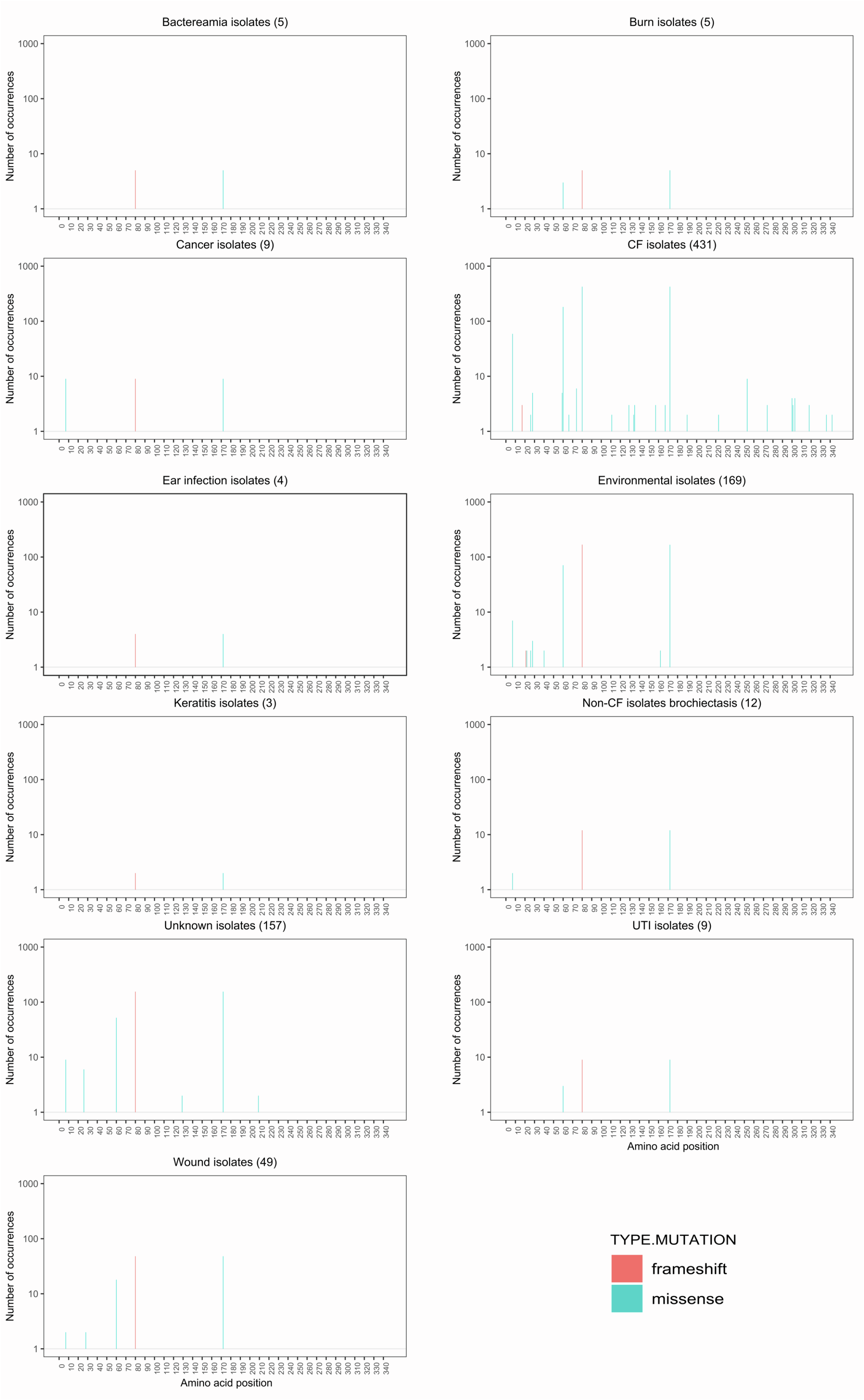
Bactereamia isolates (5)
Burn isolates (5)
Cancer isolates (9)
CF isolates (431)
Ear infection isolates (4)
Environmental isolates (169)
Keratitis isolates (3)
Non-CF isolates brochiectasis (12)
Unknown isolates (157)
UTI isolates (9)
Wound isolates (49)
Number of occurrences
1
10
100
1000
Amino acid position
0 10 20 30 40 50 60 70 80 90 100 110 120 130 140 150 160 170 180 190 200 210 220 230 240 250 260 270 280 290 300 310 320 330 340
TYPE.MUTATION
frameshift
missense

Figure S5. Distribution of mutations in MexT from diverse sources, Related to Figure 4. Each plot shows the prevalence of non-synonymous mutations in MexT in *P. aeruginosa* isolates from different isolation sources (e.g. bacteraemia, burns, environment). The different bar colours represent the type of mutation (frameshift, missense). The number in parenthesis next to the title of each chart indicates the number of isolates belonging to that category, for example, there were three keratitis isolates, and nine from urinary tract infections (UTI).

Table S1. Mutations found in by-passing mutants

Strain	Position	Mutation	Annotation	Gene	Description
**TM-62**	4,785,449	C to G	V21L	*tufB*	Elongation factor Tu
	2,807,985	G to T	V130F	*mexT*	Transcriptional Regulator MexT
**TM-79**	2,808,216	G to A	G207S	*mexT*	Transcriptional Regulator MexT
	2,990,991	Δ12 bp	coding (557-568/996 nt)	*nuoH*	NADH-quinone
**TM-83**	4,785,449	C to G	V21L	*tufB*	Elongation factor Tu
	2,807,985	G to T	V130F	*mexT*	Transcriptional Regulator MexT
	5,678,399	Δ15 bp	coding (24-38/525 nt)	*pilP*	Type 4 Fimbrial Biogenesis protein PilP
**TM-98**	4,785,449	C to G	V21L	*tufB*	Elongation factor Tu
	2,807,985	G to T	V130F	*mexT*	Transcriptional Regulator MexT
	5,070,754	G to T	G331V	*pilB*	Type 4 Fimbrial Biogenesis protein PilB

Table S2. Antimicrobial resistance profile of by-passing mutants

Strain	Mechanism of action	Antibiotic target	Antibiotic Class	Antibiotic	MIC (mg/L)	Breakpoint
**PAO1**	Protein synthesis	30S subunit	Aminoglycoside	Gentamicin	2	>4
			Tetracycline	Tetracycline	32	NA
		50S subunit	Chloramphenicol	Chloramphenicol	>512	NA
	Nucleic acid synthesis	DNA gyrase	Fluoroquinolone	Ciprofloxacin	0.5	>1
	Cell wall synthesis	PBP 2	Carbapenem	Meropenem	2	>8
		LPS	Polymyxin	Colistin	2	>2
**ΔSR**	Protein synthesis	30S subunit	Aminoglycoside	Gentamicin	2	>4
			Tetracycline	Tetracycline	32	NA
		50S subunit	Chloramphenicol	Chloramphenicol	>512	NA
	Nucleic acid synthesis	DNA gyrase	Fluoroquinolone	Ciprofloxacin	0.25	>1
	Cell wall synthesis	PBP 2	Carbapenem	Meropenem	2	>8
		LPS	Polymyxin	Colistin	2	>2
**TM-62**	Protein synthesis	30S subunit	Aminoglycoside	Gentamicin	**64**	>4
			Tetracycline	Tetracycline	16	NA
		50S subunit	Chloramphenicol	Chloramphenicol	128	NA
	Nucleic acid synthesis	DNA gyrase	Fluoroquinolone	Ciprofloxacin	0.125	>1
	Cell wall synthesis	PBP 2	Carbapenem	Meropenem	2	>8
		LPS	Polymyxin	Colistin	1	>2
**TM-79**	Protein synthesis	30S subunit	Aminoglycoside	Gentamicin	**16**	>4
			Tetracycline	Tetracycline	16	NA
		50S subunit	Chloramphenicol	Chloramphenicol	256	NA
	Nucleic acid synthesis	DNA gyrase	Fluoroquinolone	Ciprofloxacin	0.125	>1
	Cell wall synthesis	PBP 2	Carbapenem	Meropenem	2	>8
		LPS	Polymyxin	Colistin	1	>4
**TM-83**	Protein synthesis	30S subunit	Aminoglycoside	Gentamicin	**64**	>4
			Tetracycline	Tetracycline	16	NA
		50S subunit	Chloramphenicol	Chloramphenicol	128	NA
	Nucleic acid synthesis	DNA gyrase	Fluoroquinolone	Ciprofloxacin	0.125	>1
	Cell wall synthesis	PBP 2	Carbapenem	Meropenem	2	>8
		LPS	Polymyxin	Colistin	2	>2
**TM-98**	Protein synthesis	30S subunit	Aminoglycoside	Gentamicin	**64**	>4
			Tetracycline	Tetracycline	16	NA
		50S subunit	Chloramphenicol	Chloramphenicol	128	NA
	Nucleic acid synthesis	DNA gyrase	Fluoroquinolone	Ciprofloxacin	0.125	>1
	Cell wall synthesis	PBP 2	Carbapenem	Meropenem	2	>8
		LPS	Polymyxin	Colistin	2	>2

Table S3. Minimal inhibitory concentration of complemented mutants for a selection of antibiotics. Note that numbers in bold indicate an MIC above the breakpoint, meaning that they are considered resistant to that particular antibiotic.

Strain	Plasmid	Gentamicin	Ciprofloxacin	Meropenem	Colistin
PAO1	(-)	2	0.5	2	4
PAO1	pUCP20	2	0.5	2	4
PAO1	pUCP20(mexT)wt	2	2	4	2
PAO1	pUCP20(mexT)mut	2	0.5	2	4
PAO1-ΔmexT	(-)	**8**	0.125	2	**16**
PAO1-ΔmexT	pUCP20	**8**	0.125	2	**16**
PAO1-ΔmexT	pUCP20(mexT)wt	2	0.5	2	4
PAO1-ΔmexT	pUCP20(mexT)mut	**8**	0.125	2	**16**

Table S4. Strains and plasmids used.

**Strain**	**Description**	**Reference**
PAO1	Wild type PAO1 *P. aeruginosa.*	(Nguyen *et al.*, 2011)
PAO1 Δ*relA* Δ*spoT* (ΔSR)	Δ*relA* (Δ181-2019) Δ*spoT* (Δ200-1948) deletion mutant in PAO1 background.	(Nguyen *et al.*, 2011)
PAO1 ΔSR +*relA* +*spoT* (cΔSR)	Δ*relA* Δ*spoT* mutant complemented for *relA* and *spoT* genes.	(Nguyen *et al.*, 2011)
JM109 (pSB406)	BHL biosensor strain. *Escherichia coli* JM109 containing pSB406: a fusion of *rhlRI*′::*luxCDABE* on a pUC18 plasmid backbone.	(Winson *et al.*, 1998)
JM109 (pSB1075)	OdDHL biosensor strain. *Escherichia coli* JM109 containing pSB1075: a fusion of *lasRI*′::*luxCDABE* on a pUC18 plasmid backbone.	(Winson *et al.*, 1998)
PAO1 Δ*pqsA* CTX-lux::*pqsA*	PQS biosensor strain. Δ*pqsA* mutant of PAO1 containing a *pqsA* promoter :: *luxCDABE* fusion integrated at a neutral site in the chromosome.	(Fletcher *et al.*, 2007)
Escherichia coli DH5α	*Escherichia coli* laboratory reference strain.	Unknown
ΔSR-mMexT	Δ*relA* Δ*spoT* mutant with missense mutation V130F in the *mexT* gene.	This study
PAO1-mMexT	PAO1 wild type strain with missense mutation V130F in the *mexT* gene.	This study
PAO1-ΔMexT	Δ*mexT* deletion mutant in the PAO1 wild type background.	This study
**Plasmids**	**Description**	**Reference**
pTnMod-OGm	Plasmid-encoded transposon carrying Gm^R.	(Dennis and Zylstra, 1998b)
pEX19Gm	Allelic exchange plasmid (accession number KM887142).	(Hoang, *et al.*, 1998)
pEX19Gm-mMexT	pEX19Gm plasmid with missense mutant allele V130F for *mexT* gene from *P. aeruginosa* PAO1.	This study
pEX19Gm-ΔMexT	pEX19Gm plasmid with an in-frame deletion allele for *mexT* gene from *P. aeruginosa* PAO1.	This study
pUCP20	Cloning vector (accession number U07165.1).	(Olsen *et al.*, 1982)
pUCP20-*mexT*_wt	pUCP20 plasmid with *mexT* wild-type allele.	This study
pUCP20-*mexT*_mut	pUCP20 plasmid with $mexT^{V130F}$ mutant allele.	This study
1338	IPCD strain GCA_004371435 with $mexT^{R194Q}$ allele.	(Freschi, *et al.*, 2019)
442	IPCD strain GCA_003839005 with $mexT^{V199M}$ allele.	(Freschi, *et al.*, 2019)

1498	IPCD strain GCA_004371155 with *mexT*^{G207D} allele.	(Freschi, *et al.*, 2019)
1259	IPCD strain GCA_003698825 with *mexT*^{L114P} allele.	(Freschi, *et al.*, 2019)
1233	IPCD strain GCA_003833825 with *mexT*^{L199R} allele.	(Freschi, *et al.*, 2019)

Methods S1: Determination of virulence factor production, related to Figures 1 and 2.

Biofilm formation

Biofilm formation was quantified following the protocol previously reported by O'Toole *et al*. [S1]. Briefly, overnight cultures were diluted 1:100 into fresh LB, and 150 µL were added to each well in a clear-bottomed 96-well microtitre plate (Nunc, Thermo Scientific). Eight biological replicates were assessed for each strain. The plates were incubated statically for 24 h at 37˚C. The liquid culture was then removed by aspiration, and the wells were washed three times with water to remove residual planktonic bacteria. The biofilm was stained with 200 µL of 0.25% (w/v) crystal violet for 15 min at room temperature. The crystal violet solution was then removed by aspiration, and the wells were gently washed with water. The plates were dried at 37˚C for 1-2 h, and the stained attached biomass was solubilised in 200 µL of 30% (v/v) acetic acid. The absorbance of each well was measured at a wavelength of 550 nm.

Exo-proteases activity assay

Strains were grown overnight in 50 mL of lysogeny broth (LB-Lennox, Oxoid) in 250 mL flasks at 37˚C with agitation at 200 rpm. The cultures were then transferred to Falcon tubes and sedimented for 10 min at 4,000 rpm (2348 × *g*). The culture supernatants were filtered with 0.2 µm pore-size filters (Sartorius Minisart Sterile EO filters; Sartorius AG, Germany) and mixed with 1.5% (w/v) skimmed milk in a ratio 1:10 (100 µL of supernatant in 900 µL of skimmed milk). The mix was vortexed and transferred to a cuvette. The absorbance (scattering) at 600 nm was measured every 5 min for 1 h.

Swarming motility

Aliquots (2.5 µL) of an overnight culture were spotted in the centre of each swarm plate. The plates were incubated without inversion at 37˚C for 24 h, and the diameter of the swarm was measured at the widest point.

Siderophores production

The production of siderophores was assessed following the protocol previously described by Louden *et al*. [S2]. Briefly, chrome azurol S (CAS)- hexadecyltrimethylammonium bromide (HDTMA) plates were prepared. Aliquots (5 µL volume) of overnight cultures were diluted to an O.D of 1.0 and spotted onto each CAS-HDTMA plate. The siderophore(s) production was quantified from the diameter of the orange halo that formed around each bacterial colony.

Pyocyanin production

Pyocyanin concentration in the cultures was determined by following the protocol of Essar *et al*. [S3]. Briefly, 50 mL of culture was grown overnight in LB. The cells were then pelleted by centrifugation (2348 × *g*) and the supernatant was filter-sterilised (Sartorius Minisart Sterile EO filters 0.2 µm pore size; Sartorius AG, Germany). A 5 mL sample of the sterile supernatant was vigorously mixed with 3 mL of chloroform, and the phases were separated by centrifugation at 10,000 rpm (13523 × *g*) for 10 min. Three millilitres of the lower blue layer were carefully recovered and added to a new Falcon tube containing 1.5 mL 0.2 N HCl. The samples were centrifuged for 2 min at 13,523 × *g* and the upper pink layer was transferred to cuvettes. The absorbance of the solution was measured at 520 nm, and the concentration of pyocyanin per millilitre was calculated.

Detection of QS signalling molecules

Overnight cultures were pelleted (2348 × *g*) and the supernatants were filter-sterilised (Sartorius Minisart Sterile EO filters 0.2 µm pore size; Sartorius AG, Germany). QS signalling molecules (BHL, OdDHL and PQS) were detected following the protocol reported by Davenport *et al*. [Davenport et al., 2015]. Briefly, QS sensor strains [S4,S5] were grown overnight in lysogeny broth (Lennox) (Oxoid). Overnight cultures were diluted 1:100 in fresh LB and incubated at 37°C until an $O.D._{600}$ of 0.5. Sixty microliters of the cultures were added to white opaque 96-well microtitre plates (Greiner Bio-One, Germany) containing 60 µL of a 1:10 dilution of the sterile culture supernatants. The plates were incubated statically at 30°C for 3 h (BHL and PQS) or 4 h (OdDHL). Luminescence was then measured in a FLUOstar Omega microplate reader (BMG LABTECH). Measurements were carried out using biological triplicates.

Antimicrobial Susceptibility test on discs

Antimicrobial susceptibility testing was performed according to the EUCAST guidelines [S6]. Briefly, the isolates were streaked onto LB plates. Single colonies were resuspended in a sterile saline solution (0.85% NaCl) and normalised to a standard optical density (0.5 McFarland units). The solution was spread with a sterile cotton swab onto Mueller–Hinton agar plates in three different directions

(vertical, horizontal, and diagonal). The antibiotic discs (Thermo Scientific, Oxoid) were placed on the plates and incubated overnight at 37°C. Antibiotic susceptibility was determined by measuring the zone of growth inhibition, and the results were interpreted according to the EUCAST guidelines.

Determination of Minimal Inhibitory Concentration (MIC)

The MIC of different antibiotics was determined following the protocol suggested by EUCAST [S6]. Briefly, serial 1:2 dilutions of the antibiotics were prepared in Muller-Hinton broth in 96-well microtitre plates (Nunc™, Thermo Scientific™), using concentrations ranging from 0 to 512 mg/L. Overnight cultures of the indicated strains were diluted 1:100 and used to inoculate the wells (10 µL per well). The plates were sealed with parafilm and incubated statically for 16 h at 37°C. The MIC was determined as the minimal concentration of the antibiotic where no bacterial growth was observed.

References

1. O'Toole, G. A. Microtiter dish biofilm formation assay. *J. Vis. Exp.* (2011) doi:10.3791/2437.
2. Louden, B. C., Haarmann, D. & Lynne, A. M. Use of Blue Agar CAS Assay for Siderophore Detection. *J. Microbiol. Biol. Educ.* **12**, 51–53 (2011).
3. Essar, D. W., Eberly, L., Hadero, A. & Crawford, I. P. Identification and characterization of genes for a second anthranilate synthase in *Pseudomonas aeruginosa*: interchangeability of the two anthranilate synthases and evolutionary implications. *J. Bacteriol.* **172**, 884–900 (1990).
4. Winson, M. K. *et al.* Construction and analysis of luxCDABE-based plasmid sensors for investigating *N*-acyl homoserine lactone-mediated quorum sensing. *FEMS Microbiol. Lett.* **163**, 185–192 (1998).
5. Fletcher, M. P., Diggle, S. P., Cámara, M. & Williams, P. Biosensor-based assays for PQS, HHQ and related 2-alkyl-4-quinolone quorum sensing signal molecules. *Nature Protocols* vol. 2 1254–1262 Preprint at https://doi.org/10.1038/nprot.2007.158 (2007).
6. ESCMID-European Society of Clinical Microbiology & Diseases, I. EUCAST: AST of bacteria. https://www.eucast.org/ast_of_bacteria/.
